# Predictors and outcomes of stroke mimics in patients treated with intravenous thrombolysis: a single-center retrospective cohort study

**DOI:** 10.3389/fneur.2025.1629435

**Published:** 2025-12-04

**Authors:** Brigita Klimbytė, Vaidas Matijošaitis, Antanas Vaitkus

**Affiliations:** 1 Department of Neurology, Hospital of Lithuanian University of Health Sciences Kauno Klinikos, Kaunas, Lithuania; 2 Department of Neurology, Faculty of Medicine, Lithuanian University of Health Sciences, Kaunas, Lithuania

**Keywords:** stroke mimic, acute ischemic stroke, thrombolysis, adverse events, cerebrovascular disease, predictors

## Abstract

**Background:**

Intravenous thrombolysis (IVT) is most effective in acute ischemic stroke (AIS) when administered promptly; however, efforts to reduce treatment times may increase the risk of treating stroke mimics (SM). This retrospective single-center study aimed to determine the prevalence of SM among patients treated with IVT, compare them with AIS cases, evaluate the clinical implications of IVT administration, and identify possible predictors of SM.

**Methods:**

Patients who received IVT for suspected AIS between January 2022 and December 2024 were retrospectively analyzed. Only patients without early ischemic changes on non-contrast CT and without intracranial occlusion on CT angiography were included. Based on clinical presentation, imaging, and discharge diagnosis, patients were classified as AIS or SM. Data collected included demographics, clinical features, risk factors, treatment times, NIHSS scores, mRS scores, and post-IVT complications.

**Results:**

Of 724 patients treated with IVT, 330 met the inclusion criteria. Among them, 293 (88.8%) had confirmed AIS and 37 (11.2%) were SM. SM patients were significantly younger (mean age 57.4 vs. 70.2 years, *p* < 0.001), had fewer vascular risk factors, and higher rates of migraine, epilepsy, and vestibular disorders. Sensory symptoms and headache were more common in SM, whereas motor symptoms and aphasia were more frequent in AIS. Although initial NIHSS scores were comparable, SM demonstrated greater improvement at 1 and 24 h post-IVT. Functional outcomes were better in SM, with 92% achieving a discharge mRS of 0 compared to 19% in AIS (*p* < 0.001). No intracranial hemorrhage or seizures were observed in the SM group. LASSO selection and Firth’s bias-reduced logistic regression analysis identified younger age, presence of sensory symptoms, history of migraine or epilepsy, and absence of atrial fibrillation as independent predictors of SM.

**Conclusion:**

Stroke mimics accounted for 11.2% of cases treated with intravenous thrombolysis and differed significantly from AIS in clinical and demographic characteristics. IVT was safe in SM, with no major complications observed. Several clinical predictors may aid in early differentiation. However, these findings should be interpreted cautiously due to the limited number of mimic cases.

## 1 Introduction

Acute ischemic stroke (AIS) is a time-sensitive medical emergency that may be potentially treatable if intervention occurs within a limited therapeutic window. The effectiveness of intravenous thrombolysis (IVT) in AIS diminishes as the interval from symptom onset to treatment initiation increases (1, 2). Efforts to expand access and reduce the administration time of IVT for AIS may inadvertently result in treating patients presenting with non-vascular conditions mimicking stroke (3). Stroke mimics (SM) may represent approximately one-fifth of all clinically diagnosed acute ischemic stroke cases, with the proportion of mimics receiving thrombolysis reaching up to 17% (4, 5). According to the literature, stroke mimics are most frequently identified as peripheral vertigo, toxic or metabolic disturbances, seizures, complicated migraines, and functional disorders (6).

Ischemic stroke is an exclusionary clinical diagnosis in the emergency setting, typically supported by non-contrast computed tomography (NCCT), which serves as the initial imaging modality due to its widespread accessibility and rapid acquisition time (7). SM can be challenging to differentiate from AIS, particularly when initial NCCT reveals no early ischemic changes and CT angiography shows no evidence of intracranial arterial occlusion. CT perfusion imaging serves as a valuable tool not only for assessing infarct core and penumbra but also for distinguishing stroke mimics (8). However, its use is associated with certain limitations, including additional radiation exposure, potential delays in treatment, and increased procedural costs (9). For this reason, when initial NCCT and CT angiography show no significant abnormalities, clinical assessment becomes crucial in guiding the decision to administer IVT. Identifying clinical predictors of SM in this subset of patients may enhance diagnostic accuracy and facilitate early differentiation between AIS and SM during initial patient assessment.

The aim of this study was to determine the prevalence of SM among patients treated with IVT whose initial NCCT scans showed no signs of early ischemic changes and whose CT angiography revealed no evidence of intracranial arterial occlusion; to compare AIS and SM cases treated with IVT based on demographic characteristics, clinical presentations, and risk factors; to assess the clinical implications of administering IVT to SM; and to identify possible predictors of SM.

## 2 Methods

This retrospective, single-center study was conducted at the Department of Neurology, Hospital of Lithuanian University of Health Sciences Kauno Klinikos and included patients who underwent IVT within 4.5 h of symptom onset for suspected AIS between January 2022 and December 2024, whose initial NCCT showed no early signs of ischemia. All patients received intravenous alteplase (Actilyse) at a standard dose of 0.9 mg/kg (maximum 90 mg), with 10% administered as an initial bolus and the remainder infused over 60 min, according to the European Stroke Organization (ESO) and AHA/ASA guidelines.

Patients were classified as having AIS or SM based on clinical presentation, neuroimaging findings, and final discharge diagnosis. SM were defined as non-vascular conditions presenting with acute neurological deficits resembling stroke symptoms, in which subsequent clinical assessment revealed an alternative diagnosis, and diffusion-weighted magnetic resonance imaging (MRI) showed no evidence of ischemic lesions. Conversely, a diagnosis of AIS was made in patients whose history, clinical examination, and disease progression were consistent with the involvement of an intracerebral vascular territory, supported or not contradicted by brain imaging findings. Patients exhibiting signs of any intracranial arterial occlusion, including large artery, medium and distal occlusions, as well as occlusion of carotid or vertebral arteries in the neck, identified through initial CT angiography and those diagnosed with transient ischemic attack (TIA) were excluded from the analysis. TIA was distinguished from SM based on complete symptom resolution within 24 h and the absence of acute lesions on diffusion-weighted MRI. However, we acknowledge that differentiating TIA from SM is often challenging, especially in cases with transient or non-specific symptoms. In our study, final diagnostic classification was based on comprehensive clinical evaluation, imaging findings, and discharge diagnosis confirmed by the treating neurologist.

Electronic medical records were used to collect patient data, including demographics, clinical features, treatment-related factors, and outcomes. The following data were collected for both groups: gender, age, initial neurological symptoms (motor, sensory, aphasia, dysarthria, vertigo, visual abnormalities, headache, and coordination deficits), systolic and diastolic blood pressure. Neurological deficit severity was assessed using the National Institutes of Health Stroke Scale (NIHSS) at presentation, 1 h after IVT, and 24 h following IVT. Smoking, diabetes mellitus, arterial hypertension, dyslipidemia, atrial fibrillation (AF), a history of stroke or TIA, epilepsy, migraine, vestibular disorders, oncological disease, and obesity were among the risk factors and comorbidities that were recorded. Treatment-related variables included the time from symptom onset to arrival at the emergency department (ED), door-to-needle time (DTNT), onset-to-treatment time (ONT) and timing of IVT administration—categorized by time of day (8:00 a.m. to 8:00 p.m. vs. 8:00 p.m. to 8:00 a.m.) and by day of the week (weekday vs. weekend/holiday). Outcomes were assessed using the modified Rankin Scale (mRS) at baseline and at discharge. Duration of hospital stay was recorded. Complications following IVT were documented, including the incidence of post-thrombolysis intracerebral hemorrhage. Symptomatic intracranial hemorrhage was defined as radiological evidence of intracranial bleeding accompanied by clinical deterioration, indicated by an increase of ≥4 points on the NIHSS, in accordance with the ECASS III definition.

The Shapiro–Wilk test was used to assess the normality of continuous variables. Descriptive statistics are presented as mean and standard deviation for normally distributed continuous variables, median and interquartile range for non-normally distributed continuous variables, and absolute and relative frequencies for categorical variables. Between-group comparisons were performed using Student’s *t*-test or Mann–Whitney *U* test for continuous variables, depending on data distribution. The Chi-square test or Fisher’s exact test was used for categorical variables, with the latter applied when expected cell counts were less than 5. Univariate logistic regression was used to explore factors associated with stroke mimics, with odds ratios (ORs) and 95% confidence intervals (CIs) reported. To reduce overfitting due to the small number of mimic cases, a two-step modeling approach was applied. First, least absolute shrinkage and selection operator (LASSO) logistic regression was used for variable selection. All predictors were standardized (mean = 0, SD = 1) before LASSO selection. This two-step penalized regression strategy—using LASSO for variable selection and Firth’s bias-reduced logistic regression for coefficient estimation—was chosen to minimize overfitting and small-sample bias. Predictors with non-zero coefficients were then entered into a Firth’s bias-reduced logistic regression model to obtain adjusted ORs and 95% CIs. This penalized approach mitigates small-sample bias and separation effects. Model performance was assessed using the area under the receiver operating characteristic curve (AUC), accuracy, sensitivity, and specificity. Analyses were conducted in Python (scikit-learn, statsmodels). Statistical significance was set at *p* < 0.05.

## 3 Results

### 3.1 Patient characteristics

**Figure 1:**
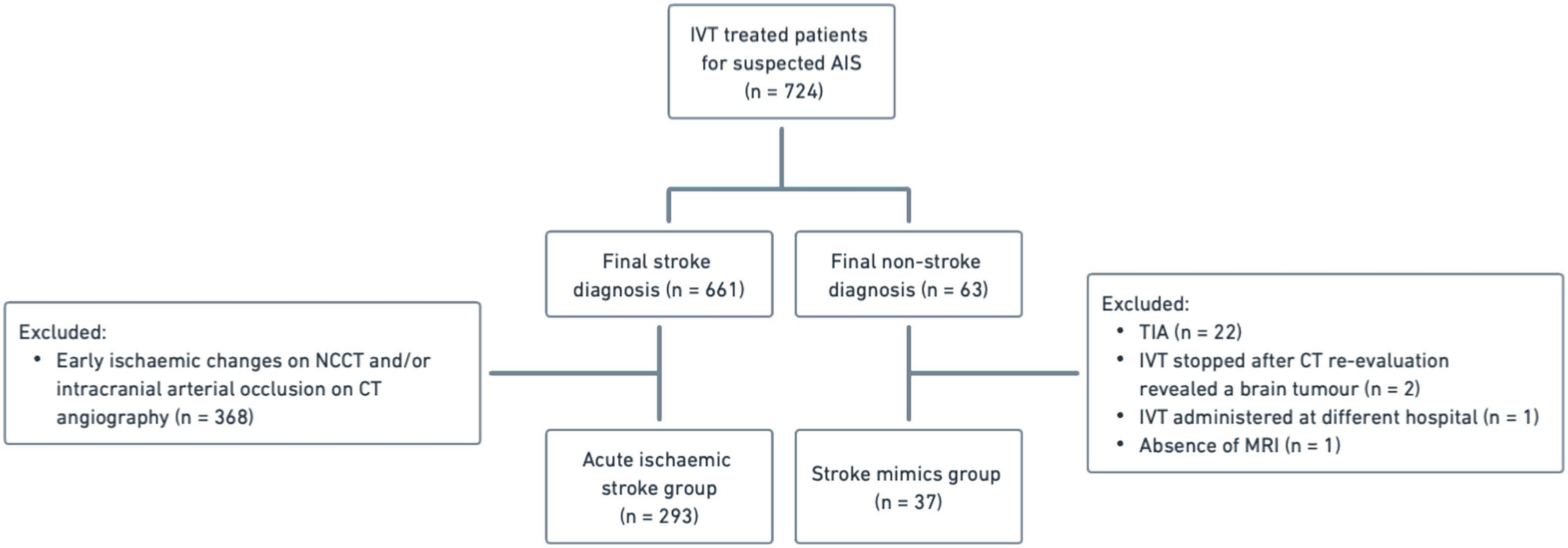
Flowchart of patient selection and enrollment for the study.

A total of 724 patients underwent IVT for suspected AIS between January 1, 2022, and December 31, 2024. Based on predefined inclusion and exclusion criteria, 330 patients were included in the final analysis, of whom 293 (88.8%) were confirmed to have acute ischemic stroke, while 37 (11.2%) were ultimately identified as stroke mimics. Figure 1 presents a detailed overview of the patient selection and enrollment process.

Table 1 presents the baseline characteristics of both groups, with groupwise comparisons. Patients with stroke mimics were significantly younger than those with ischemic stroke (mean age 57.4 ± 9.4 vs. 70.2 ± 11.6 years, *p* < 0.001). There was no significant difference in gender distribution between the groups (54% female in mimics vs. 45% in stroke, *p* = 0.32). Systolic and diastolic blood pressure measurements were similar between the groups (*p* = 0.74 and *p* = 0.65, respectively).

**Table 1:** A summary of baseline characteristics and main differences between AIS and SM groups.

Variable	Overall (*N* = 330)	Stroke (*N* = 293)	Mimic (*N* = 37)	*p*-value
Age	68.8 ± 12.1	70.2 ± 11.6	57.4 ± 9.4	<0.001*
Gender (male/female)	179/151 (54%/46%)	162/131 (55%/45%)	17/20 (46%/54%)	0.32
Initial systolic BP	161.5 ± 28.1	161.7 ± 28.2	159.9 ± 27.9	0.74
Initial diastolic BP	89.8 ± 16.6	89.9 ± 16.9	89.4 ± 14.6	0.65
Clinical presentation:				
Motor deficit (absent/present)	44/286 (13%/87%)	36/257 (12%/88%)	8/29 (22%/78%)	0.13
Motor deficit severity (no deficit/mild/severe/plegia)	44/190/62/34 (13%/58%/19%/10%)	36/169/57/31 (12%/58%/19%/11%)	8/21/5/3 (22%/57%/14%/8%)	0.55
Sensory deficit (absent/present)	124/206 (38%/62%)	117/176 (40%/60%)	7/30 (19%/81%)	0.013*
Aphasia (absent/present)	196/134 (59%/41%)	169/124 (58%/42%)	27/10 (73%/27%)	0.074
Aphasia severity (none/mild/severe)	196/105/29	169/95/29	27/10/0	0.054
	(59%/32%/9%)	(58%/32%/10%)	(73%/27%/0%)	
Dysarthria (absent/present)	235/95 (71%/29%)	208/85 (71%/29%)	27/10 (73%/27%)	0.82
Vertigo (absent/present)	286/44 (87%/13%)	257/36 (88%/12%)	29/8 (78%/22%)	0.13
Visual deficit (absent/present)	306/24 (93%/7%)	271/22 (92%/8%)	35/2 (95%/5%)	0.92
Headache (absent/present)	317/13 (96%/4%)	284/9 (97%/3%)	33/4 (89%/11%)	0.046*
Coordination deficit (absent/present)	272/58 (82%/18%)	244/48 (83%/17%)	28/9 (76%/24%)	0.3
Initial NIHSS [median (IQR)]	5 (3–7)	5 (3–7)	4 (2–6)	0.10
NIHSS after 1 h [median (IQR)]	3 (2–5)	3 (2–5)	2 (0–3)	0.001*
NIHSS after 24 h [median (IQR)]	2 (1–4)	2 (1–4)	0 (0–1)	<0.001*
Risk factors and comorbidities:				
Smoker (no/yes)	294/36 (89%/11%)	261/32 (89%/11%)	33/4 (89%/11%)	0.92
Obesity (absent/present)	311/19 (94%/6%)	277/16 (95%/5%)	34/3 (92%/8%)	0.55
Diabetes (absent/present)	269/61 (82%/18%)	234/59 (80%/20%)	35/2 (95%/5%)	0.030*
Arterial hypertension (absent/present)	59/271 (18%/82%)	47/246 (16%/84%)	12/25 (32%/68%)	0.014*
Dyslipidemia (absent/present)	172/158 (52%/48%)	151/142 (52%/48%)	21/16 (57%/43%)	0.52
Atrial fibrillation (AF) (absent/present)	271/59 (82%/18%)	234/59 (80%/20%)	37/0 (100%/0%)	0.003*
History of AF (absent/present)	286/44 (87%/13%)	249/44 (85%/15%)	37/0 (100%/0%)	0.008*
New-onset AF (absent/present)	315/15 (95%/5%)	278/15 (95%/5%)	37/0 (100%/0%)	0.4
History of stroke/TIA (absent/present)	244/86 (74%/26%)	218/75 (74%/26%)	26/11 (70%/30%)	0.61
History of epilepsy (absent/present)	325/5 (98%/2%)	291/2 (99%/1%)	34/3 (92%/8%)	0.011*
History of migraine (absent/present)	317/13 (96%/4%)	288/5 (98%/2%)	29/8 (78%/22%)	<0.001*
History of vestibulopathy (absent/present)	323/7 (98%/2%)	289/4 (99%/1%)	34/3 (92%/8%)	0.033*
History of oncology (absent/present)	297/33 (90%/10%)	261/32 (89%/11%)	36/1 (97%/3%)	0.15
Treatment characteristics:				
Time from symptom onset to arrival at ED (mean ± SD, in minutes)	108.8 ± 63.1	107.7 ± 62.8	117.3 ± 66.2	0.53
DTNT (mean ± SD, in minutes)	30.0 ± 20.3	29.4 ± 20.2	34.4 ± 21.3	0.079
ONT (mean ± SD, in minutes)	138.7 ± 64.2	137.1 ± 64.32	151.7 ± 69.0	0.20
IVT daytime (day/night)	246/84 (75%/25%)	220/73 (75%/25%)	26/11 (70%/30%)	0.58
IVT weekday (weekday/weekend)	237/93 (72%/28%)	210/83 (72%/28%)	27/10 (73%/27%)	0.91
Discharge mRS scale [median (IQR)]	1 (0–2)	1 (1–3)	0 (0–0)	<0.001*
Hospital stay (mean ± SD, days)	10.4 ± 5.8	10.8 ± 6.0	7.4 ± 3.5	<0.001*
Complication (absent/present)	290/40 (88%/12%)	254/39 (87%/13%)	36/1 (97%/3%)	0.55

Continuous variables are presented as mean (SD) and compared using Student’s *t*-test. Ordinal variables are presented as median (IQR) and compared using Mann–Whitney U test. Categorical variables are presented as n (%) and compared using Chi-squared test or Fisher’s exact test when expected frequencies were <5. *p < 0.05 indicates statistical significance.

### 3.2 Clinical presentation

Sensory symptoms were significantly more common in the mimic group compared to the stroke group (81% vs. 60%, *p* = 0.013). Headache was also more frequently reported in the mimic group (11% vs. 3.1%, *p* = 0.046). Aphasia was less common in mimics, though the difference did not reach statistical significance (27% vs. 42%, *p* = 0.074). Notably, severe aphasia was not observed in any mimic cases compared to 9.9% in the stroke group (*p* = 0.054). Motor symptoms, dysarthria, vertigo, visual changes, and coordination deficits showed no significant differences between the groups.

Among the 37 patients classified as stroke mimics, 18 cases (48.6%) simulated posterior circulation strokes, while 19 cases (51.4%) mimicked anterior circulation strokes. Table 2 presents the distribution of stroke mimics according to the vascular territory they clinically resembled.

**Table 2:** Distribution of stroke mimics by resembling vascular territory.

Vascular territory	Count	Percentage
Posterior circulation mimic	18	48.6%
Anterior circulation mimic:	19	51.4%
Right MCA^1^ territory mimic	9	24.3%
Left MCA^1^ territory mimic	10	27.0%
Total	37	100.0%

^1Middle cerebral artery.^

**Figure 2:**
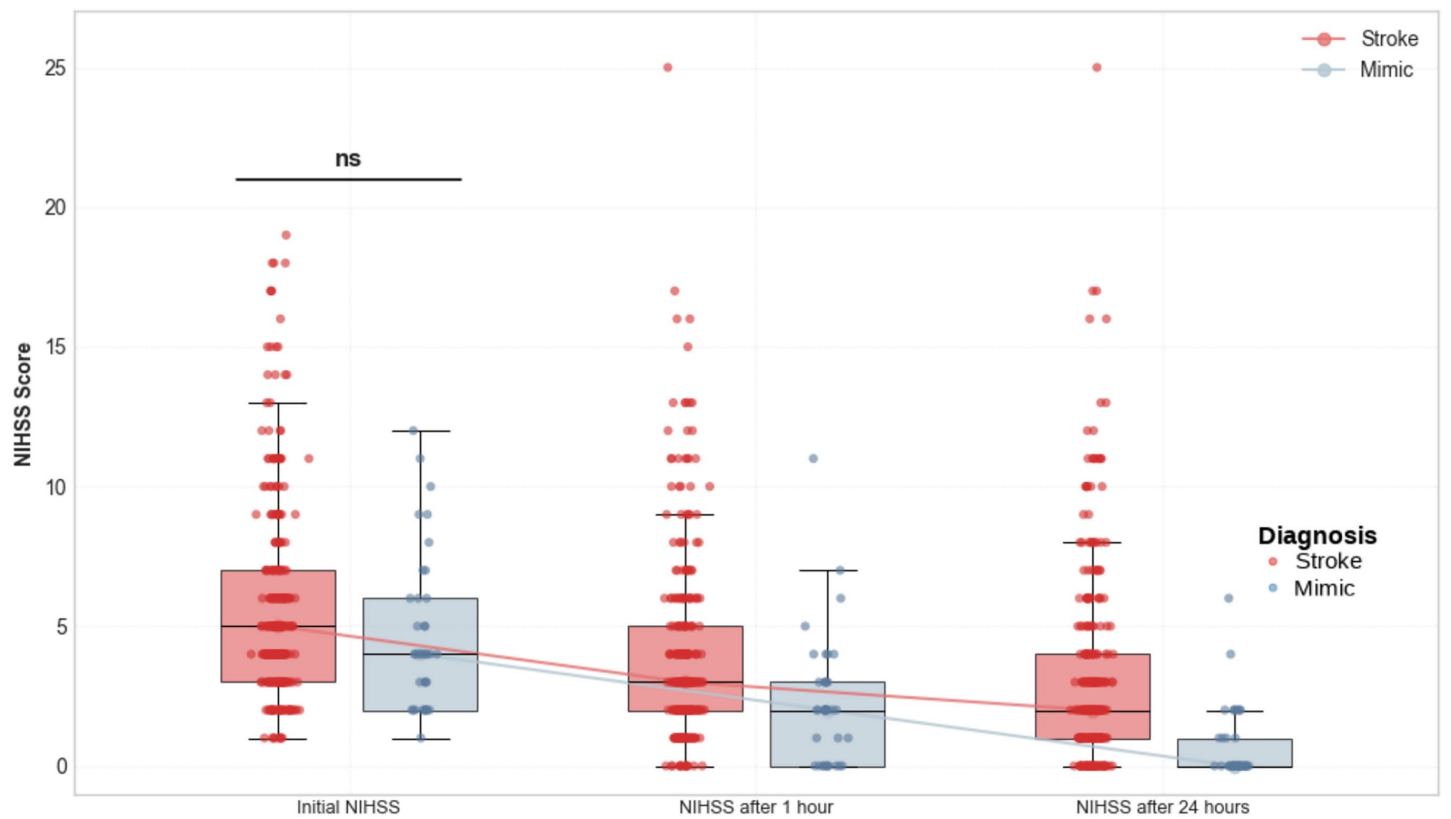
Comparison of NIHSS scores at presentation, 1 h, and 24 h after IVT between patients with AIS and SM.

The initial NIHSS scores were comparable between SM and AIS [median (IQR) for mimics 4 (2–6) vs. 5 (3–7), *p* = 0.10]. However, NIHSS scores at 1 and 24 h post-thrombolysis were significantly lower in the mimic group (2 [0–3] vs. 3 [2–5] at 1 h, *p* = 0.001 and 0 [0–1] vs. 2 [1–4] at 24 h, *p* < 0.001). Figure 2 illustrates pairwise and groupwise comparisons of NIHSS scores between the mimic and stroke groups at presentation, 1 and 24 h after the IVT.

### 3.3 Risk factors and comorbidities

Patients with stroke mimics had a significantly lower prevalence of atrial fibrillation (0% vs. 20%, *p* < 0.001), diabetes mellitus (5.4% vs. 20%, *p* = 0.030) and arterial hypertension (68% vs. 84%, *p* = 0.014). Conversely, the mimic group exhibited statistically significantly higher rates of migraine (22% vs. 1.7%, *p* < 0.001), epilepsy (8.1% vs. 0.7%, *p* = 0.011), and vestibular disorders (8.1% vs. 1.4%, *p* = 0.033). No significant differences were observed in the prevalence of smoking, dyslipidemia, history of stroke or TIA, obesity, or oncological conditions.

### 3.4 Treatment characteristics and outcomes

The time from symptom onset to arrival was similar between groups (117.3 ± 66.2 vs. 107.7 ± 62.8 min, *p* = 0.5). DTNT tended to be longer in the mimic group, though the difference was not statistically significant (34.4 ± 21.3 vs. 29.4 ± 20.2 min, *p* = 0.079). No statistically significant differences were found between groups regarding the timing of IVT administration by time of day or day of the week. Hospital stay was significantly shorter for patients with stroke mimics (7.4 ± 3.5 vs. 10.8 ± 6.0 days, *p* < 0.001).

**Figure 3:**
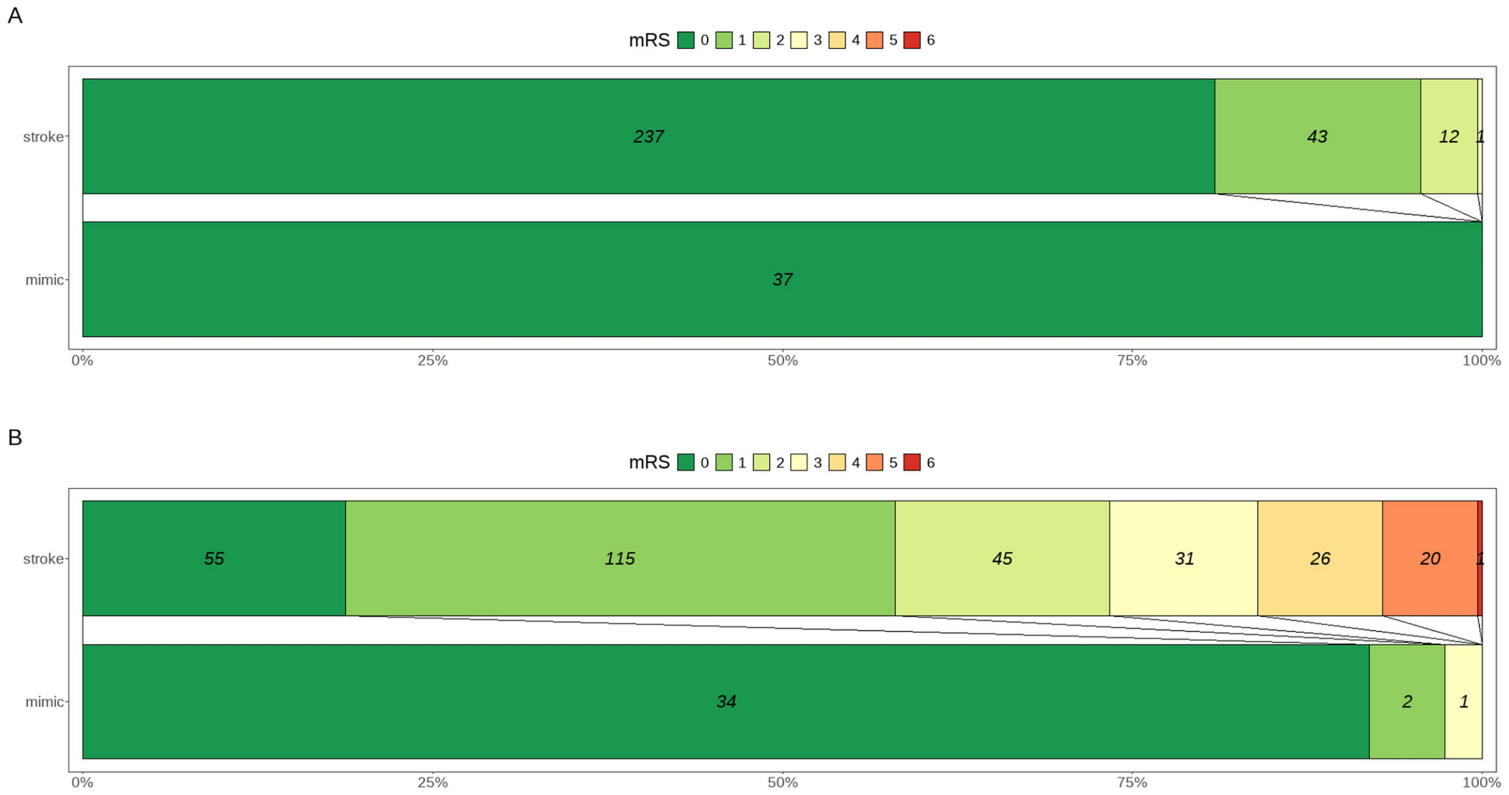
Distribution of mRS scores at presentation **(A)** and at discharge **(B)** in patients presenting with SM versus AIS.

All patients in the mimic group had a baseline mRS score of 0, compared to 81% in the stroke group (*p* = 0.013). At discharge, functional outcomes were significantly better in the mimic group, with 92% having an mRS of 0 compared to only 19% in the stroke group (*p* < 0.001). The median mRS at discharge was 0 in the mimic group versus 1 in the stroke group (*p* < 0.001). Results are visualized in Figure 3.

The overall complication rate was similar between groups, with 97% of SM and 87% of AIS experiencing no complications (*p* = 0.55). Allergic reactions were the most common complication in both groups (AIS: 4.1%, *n* = 12; SM: 2.7%, *n* = 1). Symptomatic hemorrhages (1.7%, *n* = 5), asymptomatic hemorrhages (3.1%, *n* = 9), extracranial bleeding (3.8%, *n* = 11), and seizures (0.7%, *n* = 2) occurred exclusively in AIS patients, with no such complications observed in the SM group.

### 3.5 Etiology of stroke mimics

Among the 37 patients with SM, the most common etiologies were migraine (24.3%) and functional disorders (24.3%), followed by peripheral vestibular dysfunction (16.2%), hypertensive encephalopathy (13.5%), central nervous system (CNS) tumors (8.1%), Todd’s paralysis (8.1%), cervical myelopathy (2.7%), and transient global amnesia (2.7%). Results are summarized in Table 3.

**Table 3:** Frequency distribution based on etiology of stroke mimics.

Category	Count	Percentage
Migraine	9	24.3%
Functional disorder	9	24.3%
Peripheral vestibular dysfunction	6	16.2%
Hypertensive encephalopathy	5	13.5%
CNS tumor	3	8.1%
Todd’s paralysis	3	8.1%
Cervical myelopathy	1	2.7%
Transient global amnesia	1	2.7%
Total	37	100.0%

### 3.6 Univariate analysis and factors associated with stroke mimics

Univariate logistic regression analysis identified multiple factors associated with both SM and AIS. Younger age was strongly associated with SM (OR 0.91 per year, 95% CI 0.88–0.94, *p* < 0.001). The presence of sensory symptoms (OR 2.85, 95% CI 1.28–7.25, *p* = 0.009), history of epilepsy (OR 12.8, 95% CI 2.06–100, *p* = 0.008), migraine (OR 15.9, 95% CI 4.98–55.6, *p* < 0.001), and vestibular disorders (OR 6.37, 95% CI 1.21–30.1, *p* = 0.031) were all significantly associated with increased odds of SM. Conversely, factors significantly associated with AIS included diabetes mellitus (OR 0.23, 95% CI 0.04–0.77, *p* = 0.014), atrial fibrillation (OR 0.17, CI 0.01–0.81, *p* = 0.023) and arterial hypertension (OR 0.40, 95% CI 0.19–0.87, *p* = 0.022). Additionally, there was a trend toward an association between headache at presentation and SM (OR 3.82, 95% CI 0.99–12.5, *p* = 0.051), and between aphasia and AIS (OR 0.50, 95% CI 0.23–1.05, *p* = 0.068), though these did not reach statistical significance. Results of the univariate analysis are reported in Table 4.

**Table 4:** Univariate logistic regression analysis results, indicating demographic, clinical and treatment-related factors associated with stroke mimics.

Variable	OR	95% CI	*p*-value
Age (per year)	0.91	0.88–0.94	<0.001
Gender (female)	1.45	0.73–2.92	0.28
Systolic BP	1.00	0.99–1.01	0.71
Diastolic BP	1.00	0.98–1.02	0.86
Motor symptoms (present)	0.51	0.22–1.27	0.14
Sensory symptoms (present)	2.85	1.28–7.25	0.009
Aphasia (present)	0.50	0.23–1.05	0.068
Dysarthria (present)	0.91	0.40–1.90	0.80
Vertigo (present)	1.97	0.79–4.47	0.14
Visual deficits (present)	0.70	0.11–2.53	0.63
Headache (present)	3.82	0.99–12.5	0.051
Coordination deficits (present)	1.60	0.68–3.49	0.27
Initial NIHSS (per point)	0.91	0.80–1.01	0.080
Smoking history (present)	0.99	0.28–2.69	0.98
Diabetes mellitus (present)	0.23	0.04–0.77	0.014
Atrial fibrillation (present)	0.17	0.01–0.81	0.023
Arterial hypertension (present)	0.40	0.19–0.87	0.022
Dyslipidemia (present)	0.81	0.40–1.61	0.55
Previous stroke/TIA (present)	1.23	0.56–2.55	0.59
Epilepsy (present)	12.8	2.06–100	0.008
Migraine (present)	15.9	4.98–55.6	<0.001
Vestibular disorders (present)	6.37	1.21–30.1	0.031
Oncological disease (present)	0.23	0.01–1.11	0.071
Obesity (present)	1.53	0.34–4.88	0.53
Admission time (night)	1.28	0.58–2.65	0.53
Admission day (weekend)	0.94	0.42–1.96	0.87

### 3.7 Multivariate analysis and independent factors associated with stroke mimics

LASSO logistic regression identified 10 candidate predictors with non-zero coefficients: age, gender, motor symptoms, sensory symptoms, headache, history AF, diabetes mellitus, epilepsy, migraine, and vestibular disorders. These variables were subsequently entered into a Firth’s bias-reduced logistic regression model to obtain adjusted odds ratios.

In the multivariate model, younger age (OR 0.93, 95% CI 0.89–0.96, *p* < 0.001), history of migraine (OR 8.47, 95% CI 2.08–34.47, *p* = 0.003), history of epilepsy (OR 31.11, 95% CI 2.28–424.4, *p* = 0.01), and presence of sensory symptoms (OR 3.39, 95% CI 1.31–10.2, *p* = 0.018) were independently associated with a higher likelihood of stroke mimic. Motor deficit and diabetes showed a negative trend toward association with stroke mimic (*p* ≈ 0.05–0.06) but did not reach statistical significance.

Atrial fibrillation occurred exclusively among stroke patients, producing quasi-complete separation in the data. Firth’s penalized likelihood estimation mitigated small-sample bias, but the adjusted estimate remained unstable (*β* = −8.89, SE = 44.6), precluding precise quantification of its effect. Nevertheless, the direction of association strongly supported AF as a predictor of true ischemic stroke, consistent with prior evidence.

Overall, the penalized regression model demonstrated good discriminative performance (AUC = 0.90, accuracy = 0.92, sensitivity = 0.41, and specificity = 0.98), indicating the robustness of the final set of predictors despite the limited number of stroke mimic cases. Results are summarized in Table 5.

**Table 5:** Firth’s bias-reduced logistic regression based on LASSO-selected variables identifying independent predictors of SM.

Variable	Coefficient (*β*)	SE	OR	95% CI	*p*-value
Age (per year)	−0.08	0.02	0.93	0.89–0.96	<0.001
Migraine (present)	+2.14	0.72	8.47	2.08–34.47	0.003
Epilepsy (present)	+3.44	1.33	31.11	2.28–424.44	0.01
Sensory symptoms (present)	+1.03	0.51	3.39	1.31–10.2	0.018
Atrial fibrillation (present)^1^	−8.89	44.61	<0.001	Not estimable	–
Motor symptoms (absent)	−1.08	0.56	0.34	0.11–1.02	0.055
Diabetes mellitus (absent)	−1.69	0.89	0.16	0.03–1.07	0.059

^1AF was present only among stroke patients, resulting in quasi-complete separation; penalized estimation was applied, but its effect could not be reliably quantified.^

## 4 Discussion

Ischemic stroke is a time-critical medical emergency that may be effectively treated if identified within a narrow therapeutic window. Rapid evaluation and prompt management are essential, with the decision to initiate treatment often relying on an exclusionary clinical diagnosis—particularly when non-contrast CT reveals no early ischemic changes and CT angiography shows no evidence of thrombosis. In this retrospective analysis of 330 patients who underwent IVT for suspected AIS, we found that 11.2% were ultimately diagnosed with SM. This rate is consistent with previously published data, which report SM prevalence among IVT-treated patients ranging from 2.4 to 31% (10–17). This discrepancy in the reported prevalence of SM is likely attributable to differences in the definitions used to classify SM and variations in study settings. We defined stroke mimics strictly as non-vascular conditions, excluding TIA from both AIS and SM groups. This methodological choice—adopted to isolate truly non-ischemic presentations—likely contributed to the lower observed SM prevalence compared to studies that include TIA under the mimic category (18). The differentiation between TIA and SM remains particularly complex, as both may present with transient deficits and unremarkable initial imaging. This distinction is crucial but sometimes subjective, even in MRI-confirmed cases, and represents an ongoing diagnostic challenge in hyperacute stroke triage.

It is worth noting that when evaluating all IVT-treated patients at our hospital during the study period (*N* = 724), stroke mimics accounted for 5.1% of cases. However, this proportion is significantly higher in the subgroup of patients who showed no early ischemic changes on NCCT and no arterial occlusion on CT angiography. This finding highlights that such cases represent the most diagnostically challenging group, in which accurate clinical assessment becomes critical. Identifying clinical predictors of SM in this subgroup may improve diagnostic precision and support better-informed decision-making regarding IVT administration.

In our study, the most common etiologies among SM were migraine and functional disorders (each accounting for 24.3%), followed by peripheral vestibular dysfunction (16.2%), hypertensive encephalopathy (13.5%), CNS tumors (8.1%), Todd’s paralysis (8.1%), cervical myelopathy (2.7%), and transient global amnesia (2.7%). These findings reflect the diverse clinical spectrum of stroke mimics, which encompass both neurological and systemic conditions capable of producing acute, focal neurological deficits. Our results are broadly consistent with those of Pohl et al. who, in their comprehensive review, identified seizures, migraines, functional disorders, and metabolic disturbances as the leading causes of stroke mimics (6). While seizures ranked higher in their analysis compared to our cohort, the prominence of migraine and functional etiologies in both studies highlights their central role in acute stroke-like presentations. We agree that the wide variability in the reported etiologies of stroke mimics across studies is most likely attributable to sampling bias, owing to the relatively small sample sizes commonly seen in stroke mimic studies, including our own (18). Furthermore, population characteristics such as age, sex distribution, and prevalence of comorbidities may influence mimic profiles. Our relatively high rates of migraine and functional disorders may reflect a younger demographic with fewer vascular risk factors compared to other studies. The absence of metabolic etiologies in our stroke mimic group likely reflects both pre-treatment clinical triage and the overall lower burden of systemic comorbidities in this cohort.

Our study showed that patients with SM were significantly younger than those with confirmed AIS, a finding widely supported in the literature (14–17). Additionally, SM had significantly fewer vascular risk factors, including lower rates of diabetes mellitus, atrial fibrillation, and hypertension. In contrast, conditions such as migraine, epilepsy, and vestibular disorders were more frequently observed among SM. These findings are consistent with previous studies, further reinforcing the evidence that stroke mimics are typically associated with non-vascular etiologies and a lower burden of traditional vascular risk factors (13, 18–20). While several studies have reported a higher prevalence of stroke mimics among female patients, no significant gender-related differences were observed in our study (14, 15, 17, 21).

In terms of clinical presentation, sensory symptoms and headache were more frequent among SM, while motor symptoms and aphasia were more often seen in AIS, though the latter did not reach statistical significance. Notably, severe aphasia was absent in all SM cases, which could serve as a distinguishing feature in clinical practice. These results align with previously published findings and are consistent with prior studies reporting that patients with stroke mimics are less likely to present with weakness or aphasia (15).

Although the initial NIHSS scores were similar between groups, stroke mimics demonstrated more rapid improvement at 1 and 24 h post-IVT. The mimic group also showed significantly better functional outcomes at discharge, with most patients achieving an mRS of 0, and had shorter hospital stays. From a safety perspective, IVT administration in stroke mimics appeared to be well-tolerated. No cases of symptomatic or asymptomatic intracerebral hemorrhage were observed in this group, consistent with previous reports indicating low complication rates and a favorable prognosis among mimics treated with IVT (15–18).

According to the literature, no significant difference in median DTNT has been observed between SM and AIS (14). However, in our study, the trend toward longer DTNT in SM—though not statistically significant—may reflect clinician hesitation or diagnostic uncertainty, potentially delaying the initiation of thrombolytic therapy in these patients. This highlights the need for improved predictive tools and targeted training to enhance early differentiation.

Our study found no significant difference in the prevalence of previous stroke or TIA between the SM and AIS groups. Similar findings have been reported in earlier studies, with some even suggesting a higher frequency of prior cerebrovascular events among SM (15). These results should be interpreted with caution, as previous events in the mimic group may have also been misdiagnosed as stroke or TIA.

Firth’s bias-reduced logistic regression analysis identified younger age, sensory symptoms, and a history of migraine or epilepsy as independent predictors of SM. Conversely, vascular risk factors such as AF were associated with AIS. AF was present only among patients with AIS, resulting in quasi-complete separation, but its directional association supports its established role as a strong negative predictor of SM and a major risk factor for AIS. While the events-per-variable ratio in our model was below the conventional threshold of 10 Peduzzi et al., the use of penalized regression techniques mitigated overfitting and improved estimate stability (22). The final penalized model achieved an AUC of 0.90, indicating strong discriminative ability despite the limited number of mimic events. Other variables, such as headache or aphasia, showed trends toward association with SM classification that did not reach statistical significance, likely due to limited statistical power rather than the absence of a true effect.

While our findings highlight certain clinical features that may suggest stroke mimic presentation, these predictors should complement—not replace—established thrombolysis eligibility assessments. Intravenous thrombolysis should not be withheld solely on the basis of these predictors, as the potential harm of withholding treatment in true stroke cases remains substantial.

This study has several limitations. The single-center design and inclusion of only patients without early ischemic changes on CT or arterial occlusion on CTA limit the generalizability of our findings. However, this highly selective subgroup represents the most diagnostically challenging population in acute stroke evaluation, for whom predictive tools are most needed. The retrospective design introduces inherent biases, as data were extracted from medical records, potentially limiting the accuracy and completeness of certain variables due to inconsistencies or omissions in clinical documentation. Stroke mimic classification relied in part on clinical judgment and MRI confirmation, which may introduce subjective interpretation. Furthermore, by excluding patients with vascular occlusion on CT angiography and focusing solely on those with normal initial imaging, we analyzed a more selective subgroup, which could have influenced the observed prevalence and distribution of stroke mimics. Additionally, 3-month mRS follow-up data were unavailable for the majority of cases, precluding long-term outcome analysis.

## 5 Conclusion

This study demonstrated that 11.2% of patients treated with IVT for suspected AIS—whose initial NCCT scans showed no signs of early ischemic changes and whose CT angiography revealed no evidence of intracranial arterial occlusion—were ultimately diagnosed with SM. Compared to AIS cases, SM patients were significantly younger, had fewer vascular risk factors, and more frequently presented with sensory symptoms, migraine, epilepsy, or vestibular disorders. Functional outcomes in the mimic group were excellent, and the safety profile of IVT remained favorable, with no major complications observed.

Independent predictors of stroke mimics identified in the multivariate analysis included younger age, sensory symptoms, a history of epilepsy or migraine, and absence of atrial fibrillation. These findings underscore the diagnostic challenges in acute stroke evaluation and highlight the potential value of incorporating clinical predictors to improve early differentiation and support decision-making in the emergency setting.

However, the findings should be interpreted with caution as they derive from a single tertiary center with established stroke protocols. External validation in multicenter cohorts with diverse populations and imaging availability would be valuable to confirm the generalizability of these predictors.

## Data Availability

The raw data supporting the conclusions of this article will be made available by the authors, without undue reservation.
